# Alcohol and Neurotransmitter Interactions

**Published:** 1997

**Authors:** C. Fernando Valenzuela

**Affiliations:** C. Fernando Valenzuela, M.D., Ph.D., is an instructor in the Department of Pharmacology, University of Colorado, Health Sciences Center, Denver, Colorado

**Keywords:** neurotransmitter receptors, neurotransmission, AODE (alcohol and other drug effects), AOD use behavior, AOD tolerance, AOD withdrawal syndrome, AOD craving, biological inhibition, memory, reinforcement, biochemical mechanism, literature review

## Abstract

Evidence suggests that alcohol affects brain function by interacting with multiple neurotransmitter systems, thereby disrupting the delicate balance between inhibitory and excitatory neurotransmitters. Short-term alcohol exposure tilts this balance in favor of inhibitory influences. After long-term alcohol exposure, however, the brain attempts to compensate by tilting the balance back toward equilibrium. These neurological changes occur as the development of tolerance to alcohol’s effects. When alcohol consumption is abruptly discontinued or reduced, these compensatory changes are no longer opposed by the presence of alcohol, thereby leading to the excitation of neurotransmitter systems and the development of alcohol withdrawal syndrome. Long-term alcohol intake also induces changes in many neurotransmitter systems that ultimately lead to the development of craving and alcohol-seeking behavior.

Scientists have long sought the mechanisms by which alcohol acts on the brain to modify behavior. An important finding is the demonstration that alcohol can affect the function of specific neurotransmitters^1^ (Lovinger et al. 1989). Studies of neurotransmitters and the receptors to which they bind have provided data on both the structure and the mechanism of action of these molecules as well as clues to their role in behavior. However, the function of individual neurotransmitters and their receptors cannot entirely explain a syndrome as complex as alcoholism.

Neurotransmitter systems do not function in isolation. Therefore, scientists are paying increasing attention to the integration of communication systems in the brain. Although the study of neural integration is in its infancy, enough has been learned to help guide future research. This article suggests mechanisms by which alcohol consumption may affect multiple neurotransmitter systems to influence behavior.

## Neurotransmitter Systems Work Together

Communication among neurons is organized in interacting levels. The most basic level of complexity is the arrangement of connections (i.e., synapses) between individual neurons. One neuron may connect with up to hundreds or thousands of adjacent neurons (Shepherd 1994). Each neuron releases one or a few different types of neurotransmitters. Each receptor type responds preferentially to one type of neurotransmitter. However, subtypes of the same receptor may respond differently from one another depending on the neuron or on the part of the brain in which the receptor is located. Inhibitory neurotransmitters transiently decrease the responsiveness of other neurons to further stimuli, whereas excitatory neurotransmitters produce the opposite effect. Some neurotransmitters produce longer lasting changes, contributing to processes such as learning and memory. Chemical messengers called neuromodulators modify the effects of neurotransmitters.

Successively higher levels of organization integrate the various functions of adjacent groups of neurons. At the highest level of complexity are neural pathways, sequences of neurons communicating through several brain regions (Shepherd 1994).

## Effects of Short-Term Alcohol Consumption

Short-term alcohol consumption depresses brain function by altering the balance between inhibitory and excitatory neurotransmission (see figure). Specifically, alcohol can act as a depressant by increasing inhibitory neurotransmission, by decreasing excitatory neurotransmission, or through a combination of both. Alcohol’s depressant effect on neurons may be associated with some of the behavioral manifestations of intoxication: Alcohol consumption is initially accompanied by decreased attention, alterations in memory, mood changes, and drowsiness. Continued acute consumption may result in lethargy, confusion, amnesia, loss of sensation, difficulty in breathing, and death (Draski and Deitrich 1995). Alcohol’s excitatory actions (e.g., reduction of social inhibitions) appear to be caused, at least in part, by suppression of inhibitory neurotransmitter systems (Pohorecky 1977).

**Figure:**
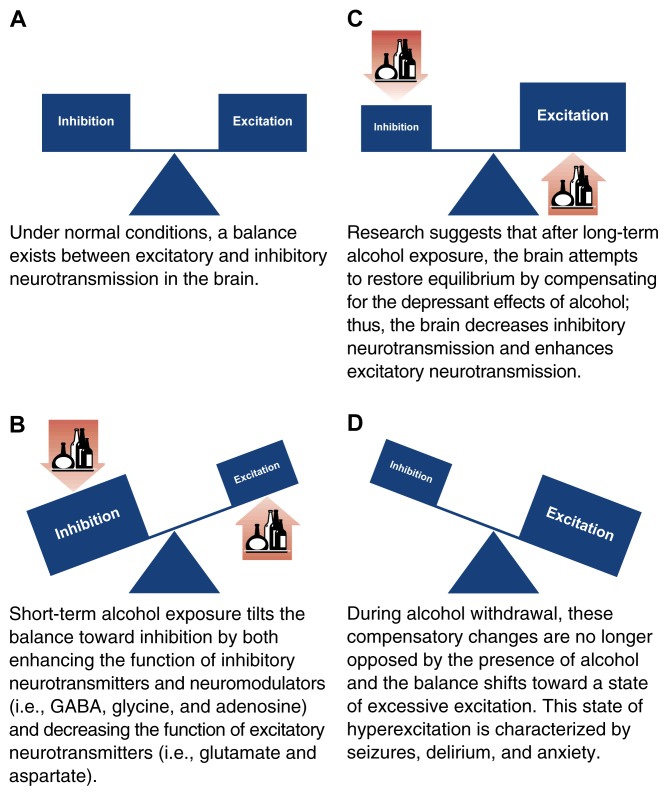
Schematic representation of alcohol’s effects on the balance of inhibitory and excitatory neurotransmission in the brain.

### Alcohol Increases Inhibitory Neurotransmission

The main inhibitory neurotransmitter in the brain is gamma-aminobutyric acid (GABA). Acting through a receptor subtype called GABA_A_, GABA leads to a state of sedation and decreased anxiety. Sedative medications such as the benzodiazepines (e.g., Valium^®^) also act at the GABA_A_ receptor. Some reports suggest that short-term alcohol exposure increases the inhibitory effect of GABA_A_ receptors (Mihic and Harris 1995). Other research, however, shows that alcohol does not increase GABA_A_ receptor function in some brain regions and under certain experimental conditions. Many factors probably determine whether GABA_A_ receptors respond to short-term alcohol exposure (Mihic and Harris 1995). Determining the mechanisms by which these factors modulate the receptor’s sensitivity to alcohol is a major focus of research.

Researchers are focusing much of their attention on other inhibitory neurotransmitters. Glycine is the major inhibitory neurotransmitter in the spinal cord and brain stem. Alcohol has been shown to increase the function of glycine receptors in laboratory preparations (Valenzuela and Harris 1997). Alcohol’s actions on inhibitory neurotransmission in this lower area of the central nervous system may cause some of alcohol’s behavioral effects.

Alcohol might also increase inhibitory neurotransmission by increasing the activity of inhibitory neuromodulators, such as adenosine. Activation of the adenosine system causes sedation, whereas inhibition of this system causes stimulation. Stimulants that inhibit the actions of adenosine include caffeine as well as theophylline, a chemical found in tea. Animal studies have shown that caffeine and theophylline reduce the sedative and motor-incoordinating effects of alcohol (Dunwiddie 1995), although these substances do not alleviate symptoms of intoxication in humans. Biochemical evidence indicates that short-term exposure to alcohol of nerve cell cultures in the laboratory increases the levels of adenosine that can interact with adenosine receptors. Thus, an alcohol-induced increase in adenosine levels might be responsible for part of alcohol’s sedative actions.

Neurotransmitter systems may interact to produce the sedative effects of alcohol. An example of such interaction occurs in Purkinje cells, a type of neuron found in the cerebellum. In these cells, the increased activation of the GABA_A_ receptor induced by alcohol occurs only with concurrent activation of certain receptors for norepinephrine, a neurotransmitter with many regulatory functions (Lin et al. 1993). Interestingly, alcohol also acts on some receptors for norepinephrine (LeMarquand et al. 1994; Tabakoff and Hoffman 1996; Valenzuela and Harris 1997).

### Alcohol Inhibits Excitatory Neurotransmission

Alcohol might induce sedative effects by reducing excitatory neurotransmission. The major excitatory neurotransmitters in the brain are the amino acids aspartate and glutamate, which act through both NMDA receptors—so named because they respond to the synthetic chemical *N*-methyl-d-aspartate—and non-NMDA receptors. Short-term exposure to intoxicating concentrations of alcohol appears to inhibit both NMDA and non-NMDA receptor activity, potentially resulting in sedation (Valenzuela and Harris 1997). As in the case of GABA_A_ receptors, however, these excitatory receptors are relatively insensitive to intoxicating concentrations of alcohol under some experimental conditions (Wright et al. 1996), underscoring the need for more research in this area.

### Investigating Alcohol’s Effects on Memory

Complex brain functions such as memory, consciousness, alertness, and learning are controlled by multiple neurotransmitter and neuromodulatory systems acting in concert. In the case of memory, researchers have postulated that information is stored in the brain as a change in the level of communication across synapses produced by an external event such as a sight or sound (Bliss and Collingridge 1993). A phenomenon called long-term potentiation (LTP) appears to be fundamental for memory formation (Bliss and Collingridge 1993). LTP is a sudden but lasting increase in the overall level of excitatory neurotransmission in the hippocampus, a brain region involved in memory. In general, LTP seems to require activation of glutamate receptors and inhibition of GABA_A_ receptors. Some studies have shown that short-term alcohol exposure inhibits glutamate receptor function (Lovinger et al. 1990) and stimulates GABA_A_ receptor function in the hippocampus (Weiner et al. 1994). Therefore, it appears that alcohol might inhibit LTP. Indeed, Morrisett and Swartzwelder (1993) reported that short-term alcohol exposure decreased LTP in the hippocampus (Bliss and Collingridge 1993). Thus, if LTP does play a role in memory storage processes, alcohol’s general inhibitory effect on memory could be related in part to its effects on glutamate and GABA systems (Weiner et al. 1997; Valenzuela and Harris 1997).

## Long-Term Alcohol Consumption

Evidence suggests that the brain attempts to restore equilibrium after long-term alcohol ingestion (see figure). For example, although short-term alcohol consumption may increase GABA_A_ receptor function, prolonged drinking has the opposite effect (Mihic and Harris 1995; Valenzuela and Harris 1997). This decrease in GABA_A_ function may result from a decrease in receptor levels or a change in the protein composition of the receptor, leading to decreased sensitivity to neurotransmission. Similarly, glutamate receptors appear to adapt to the inhibitory effects of alcohol by increasing their excitatory activity (Tabakoff and Hoffman 1996; Valenzuela and Harris 1997). Additional studies show a compensatory decrease in adenosine activity following long-term alcohol exposure (Valenzuela and Harris 1997).

### Tolerance

The compensatory changes previously described might be involved in the development of alcohol-related behavior. An example of such behavior is tolerance (i.e., a person must drink progressively more alcohol to obtain a given effect on brain function). For example, in animals exposed for several days to alcohol, many neurotransmitter receptors appear resistant to the short-term actions of alcohol on glutamate and GABA_A_ receptors compared with animals that have not been exposed to alcohol (Valenzuela and Harris 1997).

Investigators have postulated that tolerance is regulated by connections between neurons that produce multiple neurotransmitters or neuromodulators (Kalant 1993). For example, evidence indicates that vasopressin (a pituitary hormone with effects on body fluid equilibrium) plays an important role in maintaining tolerance to alcohol (Tabakoff and Hoffman 1996). Remarkably, a single exposure to a vasopressinlike chemical while an animal is under the effects of alcohol is followed by long-lasting tolerance to alcohol (Kalant 1993). The development of this long-lasting tolerance depends not only on vasopressin but also on serotonin, norepinephrine, and dopamine—neurotransmitters with multiple regulatory functions (Tabakoff and Hoffman 1996; Valenzuela and Harris 1997).

### Alcohol Withdrawal Syndrome

When alcohol consumption is abruptly reduced or discontinued, a withdrawal syndrome may follow, characterized by seizures, tremor, hallucinations, insomnia, agitation, and confusion (Metten and Crabbe 1995). Scientists postulate that this syndrome represents the hyperactivity of neural adaptive mechanisms no longer balanced by the inhibitory effects of alcohol (see figure).

Increased NMDA receptor activity significantly increases the amount of calcium that enters nerve cells. Although calcium is essential for nerve cell function, an excess of this substance within neurons has been reported to produce cell toxicity or death. In fact, repeated cycles of alcohol consumption and abstinence (e.g., binge drinking) may cause calcium-related brain damage (Hunt 1993).

GABA’s role in withdrawal is related to decreased inhibitory function. As previously noted, long-term alcohol use may lead to a decrease in GABA_A_ receptor function. In the absence of alcohol, the reduced activity of inhibitory GABA neurotransmission might contribute to the anxiety and seizures of withdrawal. These symptoms are treated, at least in part, using medications that increase GABA_A_ receptor function, such as diazepam (Valium) and other sedatives.

The GABA_A_ and NMDA receptor systems together could be responsible for a significant portion of the alcohol withdrawal syndrome. Changes in other neural systems might also be important in withdrawal, however. Voltage-sensitive calcium channels are pores in the cell membrane that admit calcium into the neuron in response to changes in electrical currents generated in the neuron.^2^ Short-term alcohol consumption inhibits calcium flow through these channels. Long-term alcohol exposure results, however, in a compensatory increase in calcium flow, which becomes excessive when alcohol consumption ceases. Evidence suggests that medications that inhibit calcium channel function (i.e., calcium channel blockers such as nimodipine) can relieve the seizures accompanying alcohol withdrawal (Valenzuela and Harris 1997).

## Reinforcement and Addiction

Reinforcement is a key phenomenon in the development of addiction to alcohol and other drugs. Positive reinforcement is the process by which an action that results in pleasure, or reward, becomes repetitive. Many people find the mental effects of alcohol consumption (e.g., euphoria) rewarding; this effect may lead to positive reinforcement and persistent alcohol-seeking behavior. The brain’s adaptive changes to the continued presence of alcohol result in feelings of discomfort and craving when alcohol consumption is abruptly reduced or discontinued. These feelings reinforce alcohol-seeking behavior during abstinence. The motivation of behavior based on avoidance of discomfort is called negative reinforcement. Both positive and negative reinforcement play a role in alcoholism (Koob et al. 1994).

Reinforcement appears to be regulated by the interaction of multiple neurotransmitter and neuromodulatory systems. Among the neurotransmitter systems linked to the reinforcing effects of alcohol are dopamine, endogenous opiates (i.e., morphinelike neurotransmitters), GABA, serotonin, and glutamate acting at the NMDA receptor (Koob 1996). Complex interactions between these neurotransmitter systems are likely to be important for the development and maintenance of alcohol-seeking behaviors. For example, alcohol has been shown to activate dopamine systems in certain areas of the brain (i.e., the limbic system) through an interaction with glutamate receptors (Koob 1996). Moreover, dopamine systems appear to be inhibited after alcohol withdrawal, and this inhibition can be reversed by alcohol consumption (Koob 1996). Interestingly, endogenous opiate systems could cause the decrease in the activity of dopamine systems that occurs during alcohol withdrawal (Koob 1996). Of particular importance regarding the role of opiate systems in alcohol reinforcement is the recent finding that opiate receptor blockers (e.g., naltrexone) reduce craving and alcohol consumption (Valenzuela and Harris 1997).

## Conclusion

Current research strongly suggests that alcohol affects multiple neurotransmitter systems in the brain. Virtually all brain functions depend on a delicate balance between excitatory and inhibitory neurotransmission. Research findings indicate that the consequences of short- and long-term brain exposure to alcohol result from alterations in this balance. However, many questions remain about the effects of alcohol on this delicate equilibrium. In addition, little is known about the molecular mechanisms of craving and addiction. Knowledge of the higher levels of neural integration is required to completely determine how alcohol affects these processes. More important, a detailed understanding of alcohol’s mechanism of action in the brain is a prerequisite to discovering effective treatments for both alcohol abuse and alcoholism.

## Footnotes

1 Nerve cells (i.e., neurons) communicate by releasing chemical messengers called neurotransmitters, which bind to receptor proteins on the surface of other neurons. This process is called neurotransmission. For definitions of technical terms used in this article, see central glossary, pp. 177–179.

2 Although neurons communicate with one another chemically, signals travel *through* a neuron in the form of an electric current.
